# Identification of Immune-Related Genes Contributing to the Development of Glioblastoma Using Weighted Gene Co-expression Network Analysis

**DOI:** 10.3389/fimmu.2020.01281

**Published:** 2020-07-16

**Authors:** Yang Kong, Zi-Chao Feng, Yu-Lin Zhang, Xiao-Fei Liu, Yuan Ma, Zhi-Min Zhao, Bin Huang, An-Jing Chen, Di Zhang, Frits Thorsen, Jian Wang, Ning Yang, Xin-Gang Li

**Affiliations:** ^1^Department of Neurosurgery, Qilu Hospital and Institute of Brain and Brain-Inspired Science, Cheeloo College of Medicine, Shandong University, Jinan, China; ^2^Shandong Key Laboratory of Brain Function Remodeling, Shandong University, Jinan, China; ^3^Department of Biomedicine, University of Bergen, Bergen, Norway; ^4^Department of Epidemiology and Health Statistics, School of Public Health, Shandong University, Jinan, China

**Keywords:** glioblastoma, macrophages, tumor microenvironment, M2 polarization, bioinformatics, TCGA

## Abstract

**Background:** The tumor microenvironment (TME) of human glioblastoma (GBM) exhibits considerable immune cell infiltration, and such cell types have been shown to be widely involved in the development of GBM. Here, weighted correlation network analysis (WGCNA) was performed on publicly available datasets to identify immune-related molecules that may contribute to the progression of GBM and thus be exploited as potential therapeutic targets.

**Methods:** WGCNA was used to identify highly correlated gene clusters in Chinese Glioma Genome Atlas glioma dataset. Immune-related genes in significant modules were subsequently validated in the Cancer Genome Atlas (TCGA) and Rembrandt databases, and impact on GBM development was examined in migration and vascular mimicry assays *in vitro* and in an orthotopic xenograft model (GL261 luciferase-GFP cells) in mice.

**Results:** WGCNA yielded 14 significant modules, one of which (black) contained genes involved in immune response and extracellular matrix formation. The intersection of these genes with a GO immune-related gene set yielded 47 immune-related genes, five of which exhibited increased expression and association with worse prognosis in GBM. One of these genes, *TREM1*, was highly expressed in areas of pseudopalisading cells around necrosis and associated with other proteins induced in angiogenesis/hypoxia. In macrophages induced from THP1 cells, *TREM1* expression levels were increased under hypoxic conditions and associated with markers of macrophage M2 polarization. *TREM1* siRNA knockdown in induced macrophages reduced their ability to promote migration and vascular mimicry in GBM cells *in vitro*, and treatment of mice with LP-17 peptide, which blocks TREM1, inhibited growth of GL261 orthotopic xenografts. Finally, blocking the cytokine receptor for CSF1 in induced macrophages also impeded their potential to promote tumor migration and vascular mimicry in GBM cells.

**Conclusions:** Our results demonstrated that TREM1 could be used as a novel immunotherapy target for glioma patients.

## Introduction

Glioblastoma (GBM) is one of the most deadly types of malignant solid tumor. Despite considerable effort toward the molecular understanding and treatment of the disease, the patient survival rate remains dismally low. The 5-year survival rate of 6.8% is especially low for GBM relative to all tumor types (1). A compounding problem for the incidence of GBM is the increasing longevity of the human population worldwide. In the United States, the incidence of GBM is estimated to be 3.22 per 100,000 individuals (1). However, the incidence of glioma rises rapidly with increasing age, reaching a peak incidence of 15.29 per 100,000 individuals in the elderly between the ages of 75 and 84 (1). Therefore, the development of effective treatment strategies to prevent the progression of GBM and improve the quality of life for patients is urgently needed.

In recent years, a molecular classification scheme adopted by the World Health Organization (WHO) has provided insight into the response of GBMs to current treatment strategies (2, 3). GBMs are now categorized as one of four molecular subtypes with variants in isocitrate dehydrogenase genes (IDH) generally appearing in cases that exhibit better overall survival. Although genetic changes reveal the precise molecular pathways corrupted during the development of individual GBMs, the biology of the brain poses additional challenges for treatment; it is a critical organ with an extremely rich blood supply, a complete blood-brain barrier (BBB), and a parenchyma lacking immune cells. Such features constitute the tumor microenvironment (TME) of GBM, which is increasingly becoming a therapeutic target of interest, in part due to the role immune cells play in tumor development.

In the last decade, checkpoint blockade immunotherapy has shown remarkable success in treating a variety of tumors, including advanced melanoma (4), non-small-cell lung cancer (NSCLC) (5), and Hodgkin's lymphoma (6). A series of clinical trials investigating the efficacy of checkpoint inhibitors in GBM showed that only a small subset of patients (8%) demonstrated objective responses (7). One possible explanation for this result is the lower tumor mutational burden of GBM (8) and the low level of T-cell infiltration (9). However, a more rigorous understanding of the biology of other immune cell types, such as tumor-associated macrophages (TAMs), which promote or inhibit the progression of GBM through the secretion of multiple cytokines (10, 11), might also provide new therapeutic targets of interest.

In this study, we performed weighted correlation network analysis (WGCNA), which identifies/generates highly correlated gene clusters by summarizing such clusters using module clustering or the identification of intramodular hub genes (12), to specifically identify immune-related genes associated with the development and/or prognosis of GBM from publicly available datasets, namely The Cancer Genome Atlas (TCGA), the Chinese Glioma Genome Atlas (CGGA), and Rembrandt. The analysis yielded a gene called *TREM1*. Inhibition of *TREM1* reduced migration and vascular mimicry *in vitro*, and tumor growth *in vivo*, possibly through decreased release of the cytokine CSF1. Thus, targeting TREM1 might be of therapeutic value in the treatment of human GBM.

## Materials and Methods

### Microarray Data

Microarray data for human gliomas were downloaded from The Cancer Genome Atlas (TCGA, http://cancergenome.nih.gov/abouttcga) (13), the Chinese Glioma Genome Atlas (CGGA, http://www.cgga.org.cn/) (14), and the Rembrandt brain cancer dataset (http://www.betastasis.com/glioma/rembrandt/) (15). These datasets include whole-genome expression profiles and corresponding clinical information of the patients.

The CGGA expression dataset was collected using the Agilent Whole Human Genome Microarray platform and includes data from a total of 301 glioma samples. All probe sets were mapped to gene symbols according to the probe annotation files of the GPL4133 platform, and gene expression values were log_2_ transformed. The TCGA and Rembrandt databases have been previously described.

### Weighted Gene Co-expression Networks and Their Modules

WGCNA is a freely accessible R software package (version R 3.4.3) developed for the construction of weighted gene co-expression networks. Rather than focusing only on differential gene expression, WGCNA uses information from the genome to identify a set of genes of interest and converts the associations of thousands of genes with phenotypes into associations between several gene sets and phenotypes, eliminating the problem of multiple hypothesis test correction. The parameter β is a soft-thresholding power parameter that strengthens strong correlations and penalizes weak correlations between genes. A hierarchical clustering tree was constructed, with different branches of the tree representing different gene modules. The adjacency matrix was transformed into a topological overlap matrix (TOM). Genes were divided into different gene modules based on the TOM-based dissimilarity measure.

### Module Genetic Analysis and Sub-network Analysis

Gene ontology (GO) (16) and Kyoto Encyclopedia of Genes and Genomes (KEGG) (17) analyses were used to explore the biological function of the module with the highest correlation with clinical traits and to screen hub genes. The STRING database (https://string-db.org/) (18) is currently the largest database of protein interactions. All genes in the selected module were first analyzed by GO and KEGG pathway enrichment analysis using DAVID web tools (https://david.ncifcrf.gov/home.jsp) (19). A plug-in for Cytoscape (20), MCODE (21), determines the hub gene and extracts sub-networks based on the degree of connectivity of genes to surrounding genes in the network. Hub genes were defined as those with gene significance (GS) > 0.3 and module membership (MM) > 0.8.

### Cell Culture and Induction of THP-1 Cell Differentiation

Human GBM cell lines U87MG and LN229 and the mouse GBM cell line GL261 were purchased from the Chinese Academy of Sciences Cell Bank (Shanghai, China). The human monocyte leukemia cell line (THP-1) was a kind gift from Professor Yuan Guo, Department of General Medicine, Shandong University. U87MG, LN229, GL261, and THP1 were cultured in Dulbecco's modified Eagle's medium (DMEM; Thermo Fisher Scientific; Waltham, MA, USA) supplemented with 10% fetal bovine serum (FBS; Thermo Fisher Scientific). THP-1 cells were treated with 200 nM phorbol-12-myristate-13-acetate (PMA; Sigma-Aldrich; St. Louis, MO, USA) for 24 h to allow for differentiation into macrophages in six-well plates. All cells were maintained at 37°C in a cell incubator containing 5% CO_2_.

### Gene Silencing

RNA interference (RNAi) technology was used to knock down the expression of target genes. Small interfering RNAs (siRNA) were synthesized (GenePharma; Shanghai, China) and transfected into cells using Lipofectamine 2000 (Thermo Fisher Scientific) according to the manufacturer's protocol. Knockdown efficiency was evaluated 48 h after transfection using RT-qPCR and Western blotting. Sequences of the siRNA *(n* = *2)* that generated efficient knockdown are the following:

si-*TREM1* 1# 5′-GGAUCAUACUAGAAGACUATT-3′; si-*TREM1* 2# 5′-GGUCAUUUGUACCCUAGGCTT-3′; si-Control: 5′-UUCUCCGAACGUGUCACGUTT-3′.

### Real-Time Quantitative PCR (RT-qPCR)

Total RNA was isolated from GBM cells using the RNA-Quick Purification Kit (Shanghai YiShan Biotechnology; Shanghai, China) according to the manufacturer's protocol. Reverse transcription was conducted using the ReverTra Ace qPCR RT Master Mix Kit (FSQ-101, TOYOBO; Osaka, Japan), and cDNA was used as the template in real-time fluorescence quantification. RT-qPCR was performed with the hot start reaction mix SYBR Green Master (Roche; Basel, Switzerland) on a Real-Time PCR Detection System (Roche 480II). Independent experiments were conducted in triplicate, and ACTB served as an internal control. The following primers were used:

TREM-1: F 5′-TTTGTTTCCCAGTCTGTGTGC-3′, R 5′-TCCCCTATTCTCCATCACCACT-3′; ACTB: F 5′-CATGTACGTTGCTATCCAGGC-3′, R 5′- CTCCTTAATGTCACGCACGAT-3′; CD206: F 5′-CGAAATGGGTTCCTCTCTGGT-3′, R 5′-TTTATCCACAGCCACGTCCC-3′; CD163: F 5′-GTAGTCTGCTCAAGATACACAGAA-3′, R 5′-GCGTTTTGAGCTCCACTCTG-3′; IL1B: F 5′-TGATGGCTTATTACAGTGGA-3′, R 5′-GGTCGGAGATTCGTAGCTGG-3′; CSF1: F 5′-CTCCAGCCAAGATGTGGTGA-3′, R 5′-TCAGAGTCCTCCCAGGTCAA-3′; CSF2: F 5′-AGCCCTGGGAGCATGTGAAT-3′, R 5′-GCAGCAGTGTCTCTACTCAGG-3′; IL6 F 5′-CCTGAACCTTCCAAAGATGGC-3′, R 5′-TTCACCAGGCAAGTCTCCTCA-3′; CXCL: F 5′-TGTGAAGGTGCAGTTTTGCC-3′, R 5′-GGGGTGGAAAGGTTTGGAGT-3′; TGF-α: F 5′-GTTGTAGCAAACCCTCAAGCTG-3′, R 5′-GAGGTACAGGCCTCTGATG-3′; VEGFA: F 5′-AAAACACAGACTCGCGTTGC-3′, R 5′-CCTCGGCTTGTCACATCTGC-3′.

### Western Blotting

Treated cell samples were lysed 30 min in RIPA buffer (Thermo Fisher Scientific) supplemented with the protease inhibitor phenylmethanesulfonyl fluoride (PMSF, Beyotime Biotechnology, Shanghai, China). Protein lysates were separated with 10% sodium dodecyl sulfate polyacrylamide gel electrophoresis (SDS-PAGE) and electrophoretically transferred to polyvinylidene difluoride (PVDF) membranes (0.22 μm, Merck Millipore; Darmstadt, Germany). Membranes were blocked at room temperature for 1 h in Tris-buffered saline with Tween-20 (TBST;10 mM Tris, 150 mM NaCl, and 0.1% Tween 20) containing 5% skim milk powder (Beyotime) and incubated overnight with primary antibody at 4°C, followed the next day by incubation with a secondary antibody conjugated to horseradish peroxidase (HRP) reconstituted in antibody dilution buffer (dilution 1: 5000; Beyotime) for 1 h at room temperature. Specific proteins were visualized with enhanced chemiluminescence (ECL, Millipore; Bedford, MA, USA) according to the manufacturer's protocol. The following primary antibodies were used: rabbit anti-TREM1 (PA5-95477, Thermo Fisher Scientific); rabbit anti-ACTB (20536-1-AP, Proteintech Group, Inc.; Wuhan, China).

### Cell Migration Assay

Transwell assays were performed in Transwell chambers (8 μm; Corning Costar; Corning, NY, USA). Cells were cultured in complete medium and supernatant with corresponding treatments (volume ratio: 1:1) for 72 h. Cells (2 × 10^4^) in DMEM medium (200 μL) were then seeded in the top chamber. The lower chamber was filled with medium (600 μL) containing 30% FBS. The chambers were incubated for 24 h. Cells that migrated to the lower surface were fixed with 4% paraformaldehyde (Solarbio; Beijing, China), stained with crystal violet (Solarbio) for 15 min, and counted under bright field microscopy (Leica DMi8; Leica Microsystems, Wetzlar, Germany). Images were acquired from 5 random fields in each well.

### Vasculogenic Mimicry (VM) Formation Assay

The VM formation assay was performed as described previously. Briefly, 96-well tissue culture plates were coated with Matrigel (0.1 mL/well; Corning; Bedford, MA, USA) and allowed to polymerize for 0.5 h at 37°C. Cells were cultured in complete medium and supernatant with corresponding treatments (volume ratio:1:1) for 72 h. Cells were resuspended, and 100 μL of suspension was seeded onto Matrigel at 2 × 10^5^ cells/mL and subsequently incubated without serum in 5% CO_2_ at 37°C for 6 h. Cultures were photographed using a Leica microscope (Leica DMi8; Leica Microsystems, Wetzlar, Germany).

### Orthotopic Xenograft Model

GL261 cells infected with lentivirus expressing luciferase-GFP cells (3 × 10^5^; OBiO Technology; Shanghai, China) were stereotactically implanted into the brains of 6-week-old C57BL/6 mice. After 7 days, tumor size was determined, and animals were divided into the following two groups: Control group, *n* = *6*, and LP-17 group, *n* = *6*. Mice were administered 50 μg of diluted control peptide (TDSRCVIGLYHPPLQVY) or 50 μg of LP-17 (LQVTDSGLYRCVIYHPP), respectively, by intravenous injection every day (GL Biochem; Shanghai, China). Tumor volume was monitored using bioluminescence imaging (PerkinElmer IVIS Spectrum; Waltham, MA, USA). At the end of the experiment, tumors were dissected and frozen in liquid nitrogen or fixed in formalin for further analysis.

### Immunohistochemistry (IHC)

Tumors were removed from sacrificed mice, fixed in 4% paraformaldehyde and embedded in paraffin. Paraffin-embedded samples were sectioned (4 μm) and fixed on glass slides. Epitope retrieval of sections was performed in 10 mmol/L citric acid buffer at pH7.2 heated in a microwave. Slides were subsequently incubated with the primary antibody (rabbit anti-CD11b, dilution 1:200, ab133357, Abcam; Cambridge, UK) at 4°C overnight followed by HRP-conjugated secondary antibody for 1 h at room temperature. Antibodies were detected using the substrate diaminobenzidine (DAB, Beyotime), and slides were counterstained with hematoxylin (Beyotime). Staining degree (scores of 0: negative, 1: light yellow, 2: light brown, and 3: dark brown) and positive ratio (scores of 1: 0–25%, 2: 26–50%, 3: 51–75%, and 4: 76–100%) were used as scoring methods for statistical analysis.

### Periodic Acid-Schiff (PAS) Stain

Briefly, slides were deparaffinized, hydrated in distilled water, immersed in PAS solution for 5 min, rinsed 4 times, incubated in Schiff's Solution for 15 min and counterstained with hematoxylin for 2–3 min (Solarbio).

### Immunofluorescence Staining

Tissue slices or VM cells were fixed with 4% paraformaldehyde at 4°C for 15 min and incubated in 0.3% Triton X-100 for 15 min. After blocking with 5% goat serum for 30 min, tissue slices or VM cells were incubated with corresponding primary antibodies against TREM1 (1:200), CD11b (1:200), CD68 (1:200), and VEGFR2 (1:200) at 4°C overnight and then incubated with Alexa Fluor 488-conjugated or Alexa Fluor 594-conjugated secondary antibodies (Beyotime) for 2 h. DAPI (Beyotime) was used to stain the nuclei. The immunofluorescent signals were detected by fluorescence microscopy (Leica DMi8; Leica Microsystems, Wetzlar, Germany). The following primary antibodies were used: rabbit anti-CD68 (ab213363, Abcam; Cambridge, UK); rabbit anti-VEGFR2 (26415-1-AP, Proteintech Group, Inc.; Wuhan, China).

### Plotting and Statistical Analysis

Each assay was performed at least three times independently. Data analysis was performed using GraphPad Prism 8 software (San Diego, CA, USA). Data were reported as the mean ± SD. The statistical significance of experimental data was evaluated using the Student's *t*-test between two groups and one-way analysis of variance (ANOVA) among more groups. A Chi-square test was used to determine the association between *TREM1* expression and pathological characteristics. The Pearson correlation was applied to evaluate the linear relationship between gene expression levels. In addition, for microarray data in a common database, non-parametric tests were used to detect differences. A log-rank model was used for single-variate survival analysis, whereas a COX regression model was used for multivariate survival analysis. Differences were considered to be significant at the following *p*-values: ^*^P < 0.05; ^**^P < 0.01; ^***^P < 0.001.

## Results

### WGCNA Identifies Key Modules in Glioma Expression Data

**Figure 1:**
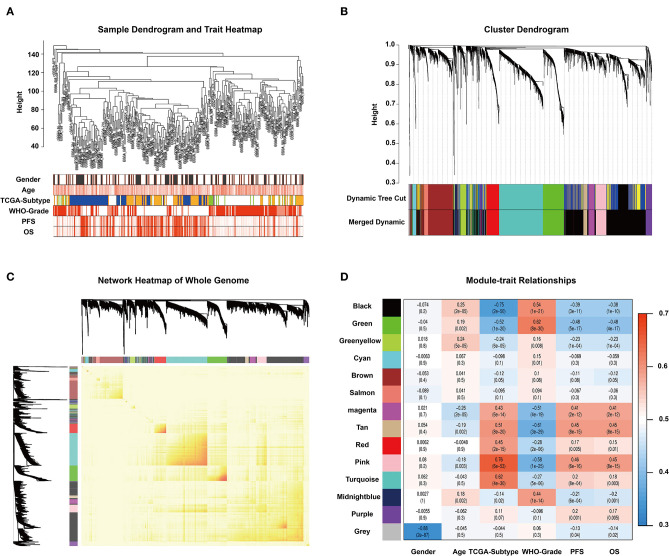
WGCNA and identification of significant modules. **(A)** Sample dendrogram and clinical trait heatmap. The cutoff value of the sample dendrogram is set to 150 to exclude samples with high variability. The samples are clustered according to clinical features (gender, age, TCGA-subtype, WHO grade, PFS, and OS). Gender: white represents male, gray represents female. Age, PFS, and OS: color depth is positively correlated with value. TCGA-subtype: blue represents neural subtype, yellow represents proneural subtype, green represents classical, and white represents mesenchymal. White, pink, and red represent WHO grades II, III, and IV, respectively. **(B)** Cluster dendrogram obtained from transcriptomic data of glioma in the CGGA database with average hierarchical linkage clustering. The color row underneath the dendrogram shows the module assignment determined by Dynamic Tree Cut and Merged Dynamic. Comparison between Dynamic Tree Cut and Merged Dynamic shows that the black and dark blue modules merge into a new black module, which means that the expression characteristics of the genes in the black module are more different. **(C)** Network heatmap of the whole genome. In the heatmap, each row and column corresponds to a gene; light color denotes low topological overlap, and progressively darker red denotes higher topological overlap. Darker squares along the diagonal correspond to modules. The gene dendrogram and module assignment are shown along the left and top. **(D)** Module–trait relationship heatmap. Hierarchical clustering of module eigengenes that summarize the modules found in the clustering analysis. The row represents the module, and the column represents the trait. The values in the box indicate the correlation and *p*-value.

To find the key modules associated with GBM clinical traits, we performed WGCNA on the CGGA glioma dataset. Clinical sample information includes gender, age, TCGA-subtype, WHO grade, progression-free survival time (PFS), and overall survival (OS). All samples were first clustered using the FlashClust package, and “150” was chosen as the criterion to exclude atypical samples (Figure 1A). The soft-thresholding power was set as “5,” and a topological matrix with non-scale features (scale-free *R*^2^ = 0.84) was obtained (Figures 1A,B). The clustering dendrograms of the sample matched the strip chart for clinical features (Figure 1B). The topological overlapping heat map depicted the TOM including all genes (Figure 1C). The topology matrix was clustered using the dissimilarity between genes and then divided into different modules. We eventually identified 14 modules (Figure 1D; non-clustering genes shown in gray). A module and sample trait correlation heatmap was created based on correlations between module eigengenes and clinical traits (Figure 1D). Finally, an eigengene adjacency heatmap showed the correlation between different modules (Figure 1C). These steps represent the general flow of analysis of expression datasets using WGCNA. Black, green, magenta, tan, and pink modules emerged as the most significant from the analysis.

### Analysis of Black Module Genes

**Figure 2:**
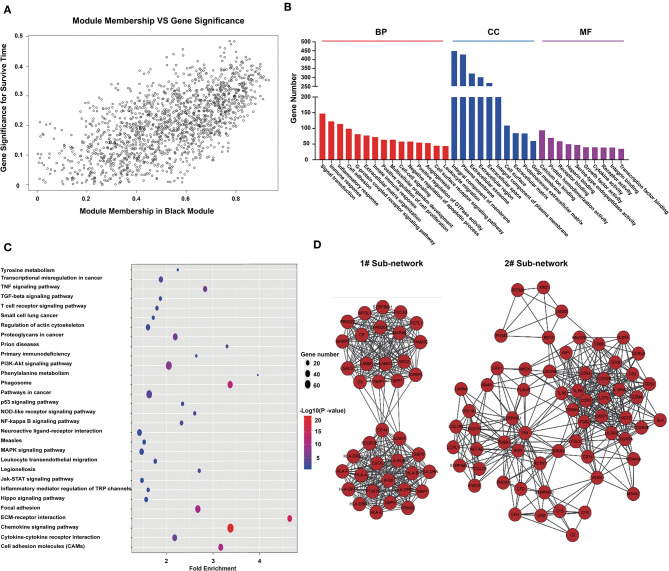
Genes in the black module are involved in immune responses and the formation of extracellular matrix. **(A)** Scatterplot of gene significance for survival time (y-axis) vs. module membership (x-axis) in the black module. The correlation coefficient between them is 0.64 (*p*-value:1.1e-175). **(B)** Histogram of GO analysis for all genes in the black module. MF, CC, and BF respectively represent molecular function, biological process, and cellular component. The FDR values of the corresponding MF items are: 5.71E-07, 1.06E-36, 9.25E-36, 0.089116, 1.8E-18, 0.007131, 6.21E-32, 4.83E-07, 0.007879, 0.001471, 9.37E-11, 2.63E-11, 2.43E-10, 0.002713, and 0.000293; CC items: 1.25E-21, 3.71E-32, 1.72E-07, 8.90E-24, 4.61E-07, 4.83E-14, 6.92E-06, 5.71E-17, 3.35E-11, and 2.63E-06; BF items: 5.64E-05, 7.84E-04, 7.04E-06, 1.82E-15, 1.51E-05, 30E-08, 1.75E-06, 6.34E-04, 0.001701, and 2.39E-09. **(C)** Bubble chart of KEGG pathway analysis for all genes in the black module. **(D)** Protein–protein network constructed by all genes in the black module. Genes were divided into some subnets based on degree of connection. The figure shows the two largest subnetworks. Most of the genes in these subnets are involved in immune responses and the formation of extracellular matrix.

From the module–trait correlations heatmap, we identified the black module as highly correlated with clinical traits (correlation coefficient = 0.64, *P* = 1.1E-175; Figure 2A). The black module, containing a total of 1,518 genes (Figure 2A), was positively correlated with the pathological grade of glioma and negatively correlated with PFS, OS, and TCGA subtypes. To reveal the potential biological functions of the genes within the black module, we conducted GO and KEGG analyses. The GO terms emerging as the most significant were biological process (BP), cellular component (CC), and molecular function (MF) (Figure 2B). GO analysis indicated that genes within the black module were mainly involved in immune response, inflammatory response, angiogenesis, cell surface receptor signaling, and leukocyte migration. KEGG pathway analysis revealed that these genes were involved in cytokine–cytokine interaction, ECM–receptor interaction, PI3K-Akt signaling, cell adhesion, and phagosomes (Figure 2C). All genes in the black module were input into String to construct a protein–protein interaction network (Supplementary Figure 2B) and then divided into several sub-networks. By setting the module membership (MM) to > 0.8 and the gene significance (GS) to > 0.3, we selected 15 hub genes from the black module: *LAMC1, LANB1, CIITA, SERPINE1, HLA-A, HLA-DBQ, IFI30, CD53, ITGB2, PTAFR, FAM20A, FN1, CCR5, LCP2, and CGR2B*. These core genes are mainly involved in the immune response and the formation of extracellular matrix. The two largest sub-networks are also shown (Figure 2D). Genes from the sub-networks are mainly involved in immune reactions, inflammatory reactions, extracellular matrix, and cell adhesion. These results led us to focus on the genes involved in the immune response in subsequent analysis.

### Identification of Immune-Related Genes

**Figure 3:**
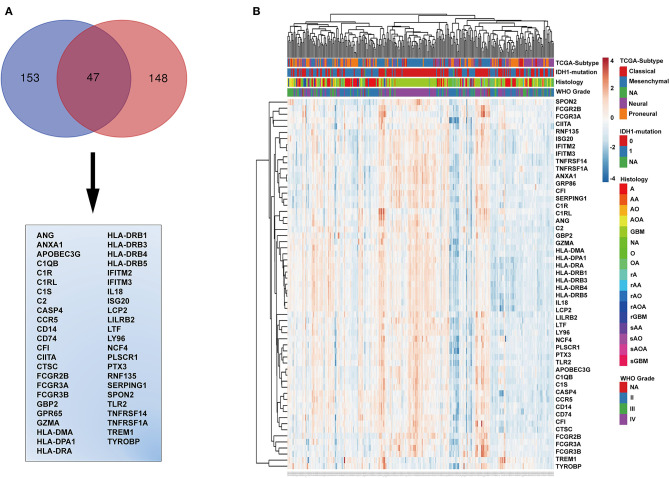
Identification of immune-related genes. **(A)** Venn diagram displaying the intersection between the top 153 genes in the black module and the top 148 immune-related genes. The 47 genes at the intersection of these two groups are listed in the table below. **(B)** Cluster heatmap generated from expression profiles and clinical information of these 47 genes from the CGGA glioma database. IDH mutation, 0, wild type; 1, mutation; NA, unknown; A, astroglioma; AA, anaplastic astrocytoma; AO, anaplastic oligodendroglioma; AOA, anaplastic oligodendroastrocytoma; GBM, glioblastoma multiforme; O, oligodendroglioma; NA, unknown; rA, recurrent astroglioma; rAA, recurrent anaplastic astrocytoma; rAO, recurrent anaplastic oligodendroglioma; rAOA, recurrent anaplastic oligodendroastrocytoma; rGBM, recurrent glioblastoma multiforme; sAA, secondary anaplastic astrocytoma; sAO, secondary anaplastic oligodendroglioma; sAOA, secondary anaplastic oligodendroastrocytoma; sGBM, secondary glioblastoma multiforme.

The genes in the black module were sorted according to their contribution to the clinical traits of the module, and the top 153 genes were selected. The intersection of these genes with GO annotation for immune-related genes (total 148 genes) yielded a list of 47 genes that are functionally involved in immunoreactions and closely related to histopathological grade, TCGA subtype, WHO grade, PFS, and OS (Figure 3A). To validate this list of 47 genes, we generated a heatmap using expression profiles and clinical data from the TCGA database (Figure 3B). These genes generally exhibited higher expression levels in *IDH* wild-type and mesenchymal molecular subtype GBMs and lower expression levels in low-grade gliomas, astrocytomas, *IDH* mutated GBMs, and neural and pro-neural molecular subtype GBMs (Figure 3B and Supplementary Figures 3A–C).

### Identification of *TREM1* as a Candidate Biomarker for Poor Prognosis

**Figure 4:**
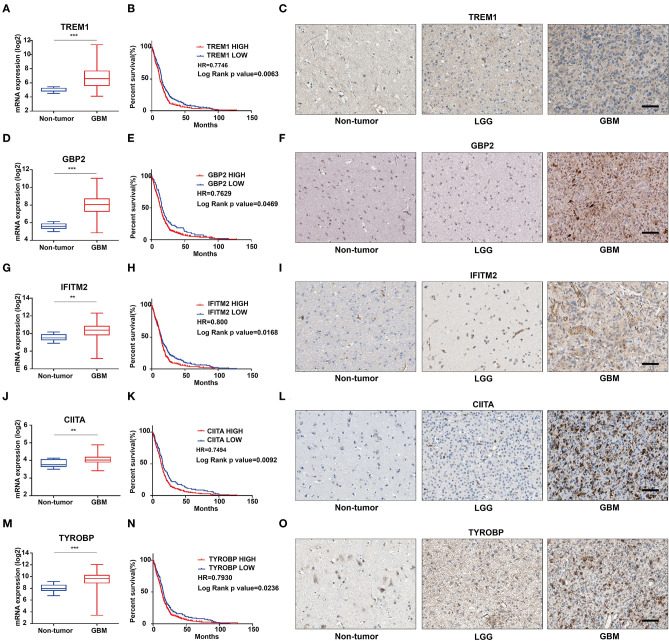
The mRNA expression, survival analysis, and protein expression of TREM1, GBP2, IFITM2, CIITA, and TYROBP using the TCGA data. The three columns consist of images for mRNA levels of genes in non-neoplastic relative to GBM tissue samples, Kaplan-Meier survival curves for patients with *gene*^*high*^ vs. *gene*^*low*^ tumor samples, and IHC for each gene in non-neoplastic, LGG, and GBM tissue samples from the TCGA database. **(A)** *TREM1* mRNA levels in non-neoplastic relative to GBM tissue samples. **(B)** Kaplan–Meier survival curves for GBM patients with *TREM1*^*high*^ and *TREM1*^*low*^ tumors. **(C)** IHC of TREM1 in non-neoplastic tissues, LGG and GBM. **(D–F)** GBP2; **(G–I)** IFITM2; **(J–L)** CIITA; **(M–O)** TYROBP. **P < 0.01; ***P < 0.001 compared to non-tumor tissue (scale bar: 100 μm).

We further characterized these 47 genes based on mRNA expression, survival prognosis, and protein expression using the TCGA GBM data. Immunohistochemistry images of antibody staining in the human protein atlas database were used to verify the protein expression of these genes (https://www.proteinatlas.org/humanproteome/pathology) (22). A group of candidate genes was chosen based on the following three characteristics: 1. mRNA expression levels were higher in GBMs than in non-tumor tissues; 2. high expression of these genes was related to worse prognosis; 3. positive IHC staining increased with increasing pathological grade of glioma (Supplementary Figures 4A–E). Genes with these characteristics included *TREM1* (Figures 4A–C), *GBP2* (Figures 4D–F), *IFITM2* (Figures 4G–I), *CIITA* (Figures 4J–L), and *TYROBP* (Figures 4M–O). Due to the fact that *TREM1* appeared prominently in the black module and little is known concerning a potential role in GBM, we mainly focused on *TREM1* for further analysis in this study.

### *TREM1* Is Associated With Poor Prognosis in All Databases

**Figure 5:**
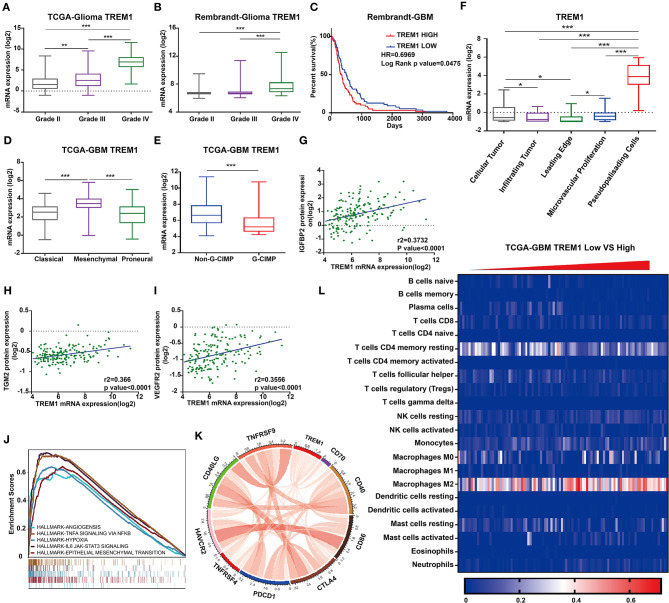
TREM1 is closely related to hypoxia and angiogenesis. *TREM1* mRNA expression profiles based on pathological grade from **(A)** TCGA-Glioma and **(B)**. Rembrandt-Glioma datasets. **(C)** Kaplan–Meier survival curves for GBM patients with *TREM1*^*high*^ and *TREM1*^*low*^ tumors using the Rembrandt database. **(D)** *TREM1* mRNA expression levels in different GBM molecular subtypes. **(E)** *TREM1* mRNA expression levels in non-GIMP and GIMP positive samples from the TCGA. **(F)** *TREM1* mRNA expression levels in different pathologic areas/tumor microsections. Corresponding data were obtained from the Ivy Glioblastoma Atlas Project. Scatter plots displaying the correlation between *TREM1* mRNA expression and protein levels of **(G)** IGFBP2, **(H)** TGM2, and **(I)** VEGFR2. **(J)** The results of representative GSEA analysis. **(K)** Chord diagram constructed from data on the correlation between *TREM1* and immune co-stimulatory factor and checkpoint molecules. **(L)** Heatmap generated with *TREM1* mRNA expression levels in multiple immune cells using the TCGA-GBM dataset. Significant difference between the two groups: *P < 0.05; **P < 0.01; ***P < 0.001.

To validate *TREM1* as a gene associated with prognosis, we examined molecular features of the gene in samples in the Rembrandt, TCGA, and CGGA databases. In all three databases, the mRNA expression of *TREM1* gradually increased with increasing WHO grade (Figures 5A,B). Furthermore, GBM *TREM1*^high^ signified a worse prognosis than GBM *TREM1*^low^ (*P* = 0.0475; Figure 5C) using the Rembrandt database. Non-G-CIMP-positive and mesenchymal molecular GBM subtype tumors expressed higher levels of *TREM1*. Many studies have demonstrated that non-G-CIMP-positive and mesenchymal molecular subtypes correlate with worse prognosis (Figures 5D,E). This result therefore indicated that expression levels of *TREM1*, the G-CIMP state, and GBM molecular subtypes may be linked. The analysis of CGGA data also verified that the expression of *TREM1* was related to gender, age, WHO grade, molecular subtype, and progression-free survival time (PFS) (Table 1). Nomograms were constructed to predict the OS of an individual patient based on a Cox proportional hazards regression model (Supplementary Table 1).

**Table 1:** Correlation of *TREM1* expression in human glioma patients with clinicopathological features.

**Variable**		**High TREMl expression**	**Low TREMl express/On**	**Chi-square values**	***P*-value**
Age	≥45	67	58	15.38	<0.0001
	<45	54	120		
Gender	Male	83	97	6.510	0.0107
	Female	38	83		
WHO	II	18	98	52.61	<0.0001
Grade	III	24	33		
	IV	76	48		
TCGA-	Classical	8	15	62.33	<0.0001
subtype	Mesenchymal	86	25		
	Proneural	19	67		
IDH1	WT	95	28	17.13	<0.0001
	Mutation	3	10		
PFS	≥643	34	114	33.27	<0.0001
	<643	82	65		

*P-values were determined by chi-square and Fisher's exact tests*.

We furthermore examined TREM1 protein expression in images of immunostained GBM samples stored in the Ivy Glioblastoma Atlas Project, which is a foundational resource for exploring the anatomic and genetic basis of GBM at the cellular and molecular levels (23). The areas of GBM samples examined (based on H&E staining) were the leading edge, infiltrating tumor, cellular tumor, microvascular proliferation, and pseudopalisading cells around necrosis. Higher expression of TREM1 appeared in areas of pseudopalisading cells around necrosis than in other regions, suggesting that TREM1 may be closely linked with hypoxia (Figure 5F).

Analysis of reverse-phase protein array data (RPPA; a high-throughput antibody-based technique) from the TCGA GBM dataset yielded proteins significantly associated with TREM1, including IFGBP2, TGM2 VEGFR2, and NDRG1, many of which have also been linked to hypoxia (Figures 5B,G–I). IGFBP2 has been reported to exert an oncogenic effect by enhancing invasiveness, angiogenesis, and VM formation and as part of a negative feedback loop with HIF1α in glioma (24–26). It has also been correlated with classic immunosuppressive biomarkers in glioma, such as *CHI3L1, TNFRSF1A, LGALS1, TIMP1, VEGFA, ANXA1*, and *LGALS3* (27). *TGM2* has been reported to be highly expressed in glioma tissues and therefore a possible diagnostic marker for glioma. *TGM2* has been shown to be related to hypoxia and *HIF1*α in malignant pleural mesothelioma and gastric cancer (28). *NDRG1*, a member of the *N-myc* downregulated gene family, is involved in stress and hormone responses, cell growth, and differentiation, and is regarded as a mesenchymal marker in GBM (29).

As one of the receptors for VEGF, VEGFR2 is a well-recognized marker for hypoxia/angiogenesis. Actually, many clinical trials using monoclonal antibodies (mAB) against the protein have been carried out in an effort to block tumor growth. However, clinical studies using bevacizumab, a humanized mAb that blocks *VEGFA* signaling, did not improve overall survival in patients with GBM (30). GBM often develops resistance to bevacizumab owing to the upregulation of alternative proangiogenic pathways and the induction of tumor cell invasion (31). Moreover, differences in angiogenic responses could originate from inter-individual GBM heterogeneity (32, 33). Although clinical results for inhibitors of VEGFR2 are inconsistent, other strategies for blocking angiogenesis might still hold promise for the treatment of GBM.

### TREM1 and Glioma-Associated Macrophages

We next performed Gene Set Enrichment Analysis (GSEA) to obtain functional profiles for molecular signatures involving *TREM1* (34). The top functional profiles were associated with angiogenesis, epithelial-mesenchymal transition, hypoxia, IL6-JAK-STAT3 signaling, TNFα signaling via NF-κb, inflammatory response, IL2-STAT5 signaling, and allograft rejection (Figures 5C,J). These results indicated that TREM1 may be induced by hypoxia and participate in angiogenesis, tumor cell migration, and other functions. This prediction has been partially confirmed in a previous work demonstrating that TREM1 was expressed on mature dendritic cells infiltrating the inflamed hypoxic joints of children affected with juvenile idiopathic arthritis. The engagement of TREM-1 elicited DAP12-linked signaling, resulting in ERK-1, Akt, and IκBα phosphorylation, and pro-inflammatory cytokine and chemokine secretion (35).

Given the vital functions of immune co-stimulatory factors and checkpoint molecules in the regulation of immune processes, we performed correlation analysis to assess the relationship between *TREM1* and several well-known genes in GBM samples. *TREM1* was correlated with *CD40, PDCD1, TNFRSF4, TNFRSF9, CD70*, and *CD86* (Figure 5K). These results corroborated a previous study demonstrating that *TREM1* was mainly expressed in tumor-associated macrophages and induced by hypoxia, thus participating in angiogenic and inflammatory responses. Importantly, TREM1 expression was not detectable in GBM cell lines under normoxia or hypoxia, indicating that TREM1 expression originated from cell types other than tumor cells (Supplementary Figures 5D,E) (36). We subsequently used TIMER, a web server for comprehensive analysis of tumor-infiltrating immune cells (https://cistrome.shinyapps.io/timer/) and found that the mRNA expression levels of *TREM1* were inversely correlated with tumor purity in GBM (37). Specifically, the mRNA expression levels of *TREM1* were negatively correlated with *CD8*-positive T-cell infiltration but positively correlated with neutrophil and dendritic cell infiltration levels (Figure 5F).

We also used Cibersort (https://cibersort.stanford.edu/) to further explore the relationship between *TREM1* and immune cell infiltration. Cibersort converts gene expression profile data into relative quantification of immune cells (38). We divided the TCGA GBM data into high- and low-expression groups based on the median expression level of *TREM1*, quantified immune cell populations, and plotted these results in heatmaps (Figure 5K). We found that the percentage of M2 macrophages increased significantly in GBM samples with high expression of *TREM1*. Thus, we proposed that high expression of *TREM1* plays a role in promoting the development and progression of gliomas, similar to the mechanism of M2 macrophages in promoting the disease. We therefore performed a series of experiments *in vivo* and *in vitro* to test this hypothesis.

### TREM1 Promotes GBM Cell Migration and VM Formation

**Figure 6:**
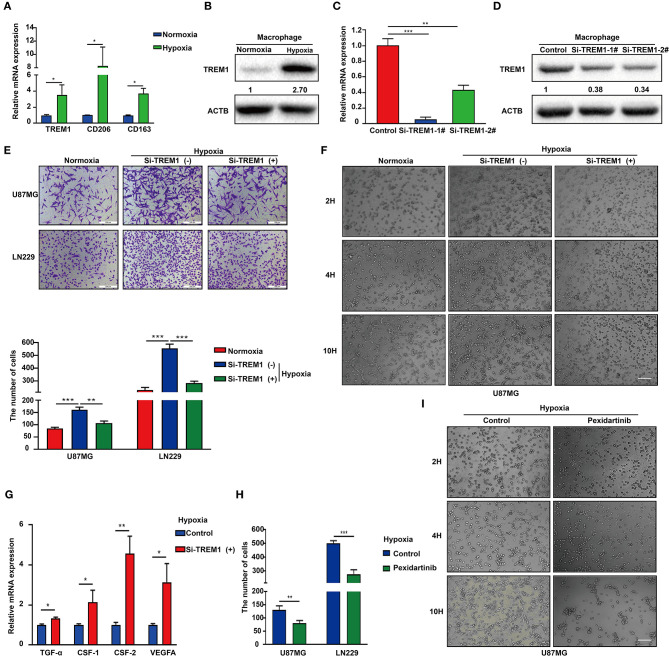
Inhibition of TREM1 suppresses migration and vasculary mimicry formation of GBM cells *in vitro*. **(A)** RT-qPCR results for *TREM1, CD206*, and *CD163* in macrophages derived from the THP1 cell line under normoxia and hypoxia. **(B)** Western blot analysis of TREM1 protein levels under normoxia and hypoxia. **(C)** Graphical representation of qRT-PCR after siRNA knockdown of TREM1. *ACTB* was used as an internal reference. **(D)** Western blot to validate the efficiency of si-TREM1 knockdown in macrophages derived from the THP1 cell line. ACTB was used as a protein loading control. **(E)** Representative images of Transwell migration for U87MG and LN229 under normoxia, hypoxia plus control, and hypoxia plus si-TREM1 (scale bar: 200 μm). Statistical results of the invasive ratio with corresponding treatment for 24 h in the Transwell assay. **(F)** Representative images of VM formation assay for U87MG in normoxia, hypoxia plus control, and hypoxia plus si-TREM1. **(G)** RT-qPCR to detect mRNA expression of *TGF-*α, *CSF1, CSF2*, and *VEGFA* after knockdown of TREM1 under hypoxia. **(H)** Statistical results of the invasive ratio for U87MG and LN229 in both control and pexidartinib (10 μM) treatment under hypoxia. **(I)** Representative images of VM formation assay for U87MG in both control and pexidartinib (10 μM) groups under hypoxic conditions. Significant difference between the two groups: *P < 0.05; **P < 0.01; ***P < 0.001.

Hypoxic necrosis is a major feature in the diagnosis of GBM and is closely related to stem cell maintenance, angiogenesis, energy metabolism, and growth characteristics of tumor cells. Based on the literature and our previous analysis, we hypothesized that *TREM1* may be induced by hypoxia. We also used immunofluorescence to confirm that *TREM1* was mainly found to be expressed in macrophages (Figure 6A). *TREM1* expression levels were increased in macrophages cultured under hypoxic conditions relative to those cultured under normoxic conditions. Markers of macrophage polarization, *CD206* and *CD163* (markers of M2 polarization), were also elevated under hypoxic conditions (Figures 6A,B).

Supernatants from hypoxic M2 macrophages have been shown to promote the proliferation of pulmonary artery smooth muscle cells (39). Inhibition of GTP cyclohydrolase (GCH1) was discovered to shift the phenotype of TAMs from proangiogenic M2 toward M1, accompanied by a shift in plasma chemokines (40). Host-produced histidine-rich glycoproteins have also been found to inhibit tumor growth and metastasis while improving the effects of chemotherapy by skewing TAM polarization away from M2 to a tumor-inhibiting M1-like phenotype (41). *TREM1* has been shown to act as an inflammatory amplifier, specifically releasing pro-inflammatory chemokines and cytokines or altering the expression of activated cell membrane surfaces upon receipt of external stimuli. Therefore, we suspect that increased *TREM1* may play a role in promoting tumor migration and angiogenesis through the release of certain inflammatory factors. We therefore knocked down *TREM1* with siRNAs in THP1 cells induced to become macrophages and examined their role in promoting biological properties of GBM cells such as migration. Western blot and qRCR analysis were used to verify the knockdown efficiency of *TREM1* (Figures 6C,D). After induction of siRNA-treated THP1 cells into macrophages, they were cultured for 24 h under normoxia and hypoxia, and supernatants were collected and mixed 1:1 with culture media containing 10% FBS for incubation with GBM cells.

The results of Transwell and VM formation assays demonstrated that hypoxia-induced macrophages promoted U87 and LN229 tumor cell migration and vascular mimicry but that this effect was significantly reduced after knockdown of *TREM1* (Figures 6B,E,F). To explore the molecular mechanism, we compared the changes in expression levels of critical cytokine mRNAs in macrophages under normoxic and hypoxic conditions as well as between control and *TREM1* knockdown groups. Analysis of the intersection of significantly altered genes in these two groups yielded *CSF1* as a common factor potentially involved in *TREM1* (Figures 6C,G). To confirm that CSF1 plays a role in promoting GBM cell migration and vascular mimicry, induced macrophages were exposed to pexidartinib (10 μM), an inhibitor of the CSF1 receptor (CSF1R). Supernatants from pexidartinib-treated macrophages relative to controls significantly inhibited cell migration and pathological angiogenesis under hypoxic conditions (Figures 6D,H,I). These results indicated that hypoxia can induce upregulation of the expression of *TREM1* in macrophages, thereby promoting GBM progression through the release of CSF-1, which triggers invasion and vascular mimicry in GBM cells.

### *TREM1* Contributes to GBM Progression *in vivo*

**Figure 7:**
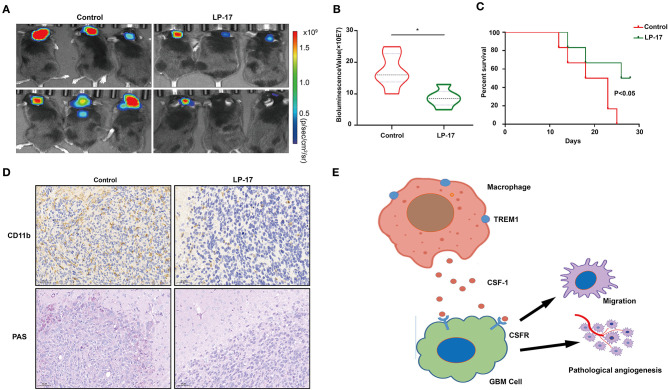
Inhibition of TREM1 suppresses tumor growth *in vivo*. **(A)** Representative bioluminescence images of tumor-bearing mice from control and LP-17 treatment groups at week 3. **(B)** Fluorescence quantitation for xenografts in animals from both groups at week 3. **(C)** Kaplan-Meier survival curves for tumor bearing animals in control and LP-17 treatment groups. A log-rank test was used to assess the statistical significance of the differences. **(D)** Images of IHC staining for CD11b and PAS staining in tumors from each group as indicated (scale bars: 50 μm). **(E)** A hypothetical schematic diagram depicts how TREM1 promotes the development of GBM. In hypoxic conditions, macrophages express increased levels of TREM1 and release more CSF1, which promotes pathological angiogenesis and migration of GBM cells. *P < 0.05 compared to control.

To investigate the effect of *TREM1* on tumor growth *in vivo*, we treated mice bearing orthotopic GBM xenograft with peptides as a control and with LP17, which blocks TREM1. The results from bioluminescence imaging demonstrated that tumor growth was inhibited in animals treated with LP17 relative to controls (~ 16.3 × 10^7^ vs. ~ 8.2 × 10^7^ photons/s, control vs. LP17-treated; Figures 7A,B). The OS of tumor-bearing animals was enhanced under treatment with LP17 compared to controls (median survival, > 28 days vs. 20.5 days, LP17 and control peptide, respectively, *P* < 0.05) (Figure 7C). Immunostaining for CD11b positive cells in the LP17-treated group showed a dramatic decrease compared to the control group (Figure 7D). Sections stained with PAS showed that in the LP17 treatment group, vascular mimicry was decreased compared to controls. In summary, these data demonstrate that inhibition of *TREM1* blocked the progression of GBM *in vivo* and may be used as a therapeutic target.

## Discussion

The current standard of care for GBM includes surgery, radiotherapy, and chemotherapy (temozolomide, TMZ). New complications arise with each arm of this multi-modal treatment, and tumors recur not long after the primary diagnosis. The most promising approach in recent years for other tumor types has been immunotherapy. Our present work also supports the possibility of interfering with alternative immune cell types typically infiltrating GBM. However, immunotherapy has not proven satisfactory for the clinical treatment of GBM. For example, although immune checkpoint inhibitors, such as anti-programmed cell death (PD)1 antibody, have achieved better prognosis in GBM animal models, a recent clinical trial indicated that PD-1 inhibitors have an objective response rate of only 8% in patients with recurrent GBM (7). In another approach, CAR-T therapy has achieved tremendous success in hematological malignancies. However, CAR-T therapy targeting EGFRvIII, a tumor-specific antigen, has not achieved the desired clinical results in GBM treatment (42).

Several possibilities might account for the reduced efficacy of immunotherapy in GBM treatment. First, the immunocompetent mouse models used to study immunotherapy do not accurately reflect the human GBM TME. The methylcholanthrene-induced GL261 and SMA-560 models are the commonly used orthotopic xenograft models in GBM immunotherapy (27). However, both model types possess a high number of mutations and predict neoepitopes and enhanced immune cell infiltration. These features are in contrast to primary GBM samples, which typically exhibit a low tumor mutational load and an immunosuppressive microenvironment (8). Second, immune cell infiltration is significantly less than in other solid tumors, rendering GBM a so-called “cold” tumor. However, immune checkpoint inhibitors still exert anti-GBM effects even though they rely on the recovery of reactive T cells to execute a killing effect (9). Third, although CAR-T therapy generates a significant increase in the number of killer T cells, the presence of the BBB may limit their access to brain tumors, unlike for other solid tumors. Moreover, even after entering the tumor microenvironment, killer T cells may have reduced killing potential due to hypoxic conditions generated because of *IDH* variants and the heterogeneity of *EGFR* mutations (7). Fourth, in addition to PD-L1, PD-1, and CTLA-4, TMEs of GBM may also contain other immunosuppressive factors, such as the A2aR high-affinity adenosine receptor (on lymphocytes and tumor-associated macrophages) or PD-L2 (on macrophages lacking PD-L1 expression). It has been reported that anti-PD-L1 and anti-TIGIT (a novel immune checkpoint inhibitor) combination therapy improved overall survival in GBM patients by increasing effector T cell function and downregulating the number of suppressive Tregs and tumor-infiltrating dendritic cells (43).

In addition, both immune checkpoint inhibitors and CAR-T therapy rely on killer T cells, but the GBM TME exhibits mass macrophage infiltration, which includes phenotypically suppressive CD163+ M2 to undifferentiated M0 macrophages, particularly in the mesenchymal molecular GBM subtype (30). Our laboratory has reported that hypoxic glioma-derived exosomes deliver microRNA-1246 to induce M2 macrophage polarization, which promotes proliferation, migration, and invasion *in vitro* and *in vivo* of glioma cells by targeting telomere binding repeat 2 interacting protein (TERF2IP) through the STAT3 and NF-κB pathways (44). It was also found that M2 macrophages enhance phosphoglycerate kinase 1 (PGK1) threonine 243 phosphorylation, which facilitates glycolysis, proliferation, and tumorigenesis in GBM cells (10). In the present study, we also found that the expression levels of *TREM1* may be accompanied by an increase in macrophage M2 polarization. This result contradicts previous indications that *TREM1* is an M1 marker of macrophages in liver biopsies. The reason for this discrepancy may be due to pathological and tissue differences.

Several other studies support a role for *TREM1* in the development of cancer. *TREM1* has been reported to exert pro-inflammatory immune responses not only in acute pathogen-induced reactions but also in chronic and non-infectious inflammatory disorders, including various types of cancer. *TREM1*-/- mice exhibited reduced tumor number and load in an experimental model of inflammation-driven tumorigenesis of colorectal tumor (45). *TREM1* has also been reported to cooperate with diminished DNA damage response *in vivo* to promote expansion and leukemic progression in Fanca-/- pre-leukemia stem cells (46). Our present study demonstrated that increased expression of *TREM1* in macrophages may promote GBM progression through the release of CSF1. The CSF1 receptor (CSF1R) has been investigated as a possible therapeutic target in the treatment of GBM. Inhibition of CSF1R has been shown to alter the expression of activated M2 markers and to reduce intracranial growth of patient-derived glioma xenografts (47). CSF1R ligand expression was also found to be elevated in GBM xenografts treated with ionizing radiation (48). Both studies indicate that inhibition of CSF1R might be a promising strategy to improve the treatment and prognosis of GBM.

Besides *TREM1*, other genes may also have potential therapeutic roles in GBM treatment. For instance, *GBP2* was found to inhibit mitochondrial fission and cell metastasis in breast cancer cells both *in vitro* and *in vivo* (49). Further, *IFITM2* was significantly up-regulated and induced after activation of beta-catenin signaling in colorectal cancers (50), and it was also reported to promote gastric cancer growth and metastasis through the insulin-like growth factor (IGF1)/IGF1 receptor (IGF1R)/STAT3 signaling pathway (51). *CIITA*, a member of the interferon response factor (IRF) pathway, was found by integrative genomic analysis to be a key oncogenic gene in primary mediastinal large B-cell lymphoma (52). *TYROBP*, a downstream effector of *TREM1*, induced the transformation of microglial cells and regulated inflammatory response (53). Thus, all of these genes will be highly interesting for studies of GBM.

In summary, we analyzed the GBM data in the CGGA database using WGCNA to obtain immune-related genes that may promote the progression of GBM. *TREM1* emerged as a gene of interest due to higher expression in GBMs relative to non-neoplastic tissue and association with a worse prognosis. The expression of *TREM1* increased in macrophages under hypoxia, and supernatants from these cells promoted pathological angiogenesis and migration of GBM cells *in vitro*. A possible factor mediating this response is CSF1 (Figure 7E). These results underscore the importance of the TME in GBM development. Thus, targeting the tumor microenvironment, or specifically TAMs, allow a vulnerability in the development of GBM to be exploited and should be considered as a viable therapeutic strategy.

## Data Availability Statement

Publicly available datasets were analyzed in this study. This data can be found here: http://cancergenome.nih.gov/abouttcga, http://www.cgga.org.cn/, http://www.betastasis.com/glioma/rembrandt/.

## Ethics Statement

The animal study was reviewed and approved by the Institutional Animal Care and Use Committee (IACUC) of Shandong University.

## Author Contributions

YK, Z-CF, NY, and X-GL conceived the study. YK and NY were involved in bioinformatics analysis. YK and Z-CF performed experiments. Y-LZ, YM, Z-MZ, and DZ participated in animal experiments. A-JC and BH performed the statistical analysis. YK and Z-CF drafted the paper. X-FL and JW supplemented manuscript. FT, JW, NY, and X-GL contributed substantially to its revision. NY and X-GL supervised the study. All authors read and approved the final manuscript.

## Conflict of Interest

The authors declare that the research was conducted in the absence of any commercial or financial relationships that could be construed as a potential conflict of interest.

## Supplementary Material

Supplementary Figure 1 **(A)** Network topology for different soft-thresholding powers. Numbers in the plots indicate the corresponding soft thresholding powers. The approximate scale-free topology can be attained at a soft-thresholding power of 5. **(B)** Assessing the scale-free topology when the soft-thresholding power was set to 5 (scale-free *R*^2^ = 0.84, slope = −1.35). **(C)** Eigengene adjacency heatmap. Different colors indicate the degree of correlation between modules.

Supplementary Figure 2 **(A)** Heatmap for gene expression in black modules. The average expression of the eigenvectors of the black module is highly correlated with the expression of genes within the black module. **(B)** Protein–protein interaction network consisting of all genes in the black module.

Supplementary Figure 3 The expression of intersection genes in different groups based on the CGGA database. **(A)** Expression of IDH wild-type and mutant. **(B)** Expression of LGG and GBM. **(C)** Expression of the non-mesenchymal subgroup and mesenchymal subgroup.

Supplementary Figure 4 Quantitative graph of IHC staining in normal tissue and glioma samples. **(A)** TREM1; **(B)** GBP2; **(C)** IFITM2**; (D)** CIITA; and **(E)** TYROBP. Significant difference between the two groups: ^*^*P* < 0.05; ^**^*P* < 0.01; ^***^*P* < 0.001.

Supplementary Figure 5 **(A)** *TREM1* mRNA expression levels in both LGG and GBM samples from the CGGA database. **(B)** Scatter plot displaying the correlation between *TREM1* mRNA expression levels and NDRG1pT346 protein levels. **(C)** GSEA highlighting a positive association of increased *TREM1* expression levels with inflammatory response, IL2-STAT5 signaling, and allograft rejection. **(D)** Immunofluorescence staining of GBM tissue sections. Red represents TREM1, blue represents DAPI (scale bar: 100 μm). **(E)** Western blot analysis of TREM1 protein levels in three GBM cell lines under normoxia and hypoxia. **(F)** The correlation between *TREM1* mRNA expression and immune infiltration levels in GBM. These images were generated using TIMER.

Supplementary Figure 6 **(A)** Immunofluorescence staining of GBM tissue sections. Red represents TREM1, green represents CD11b and CD68, respectively, and blue represents DAPI (scale bar: 100 μm). **(B)** Immunofluorescence staining of VM for U87MG under hypoxia condition plus control or si-TREM1. Green represents VEGFR2, and blue represents DAPI (scale bar: 100 μm). **(C)** mRNA expressions of *TGF-*α, *IL1*β, *IL6, IL10, CSF1, CSF2, CXCL*, and *VEGFA* were detected under normoxia and hypoxia. **(D)** Representative images of Transwell migration for U87MG and LN229 in both control and pexidartinib treatment under hypoxia (scale bar: 200 μm). Significant difference between the two groups: ^*^*P* < 0.05; ^**^*P* < 0.01; ^***^*P* < 0.001.

Supplementary Table 1 Nomogram for predicting the proportion of glioma patients with OS based on the TCGA database.
