# Supplementary figures and images for: Identification of Immune-Related Genes Contributing to the Development of Glioblastoma Using Weighted Gene Co-expression Network Analysis
Source: Front Immunol. 2020 Jul 16;11:1281. doi: 10.3389/fimmu.2020.01281 (PMC7378359; doi:10.3389/fimmu.2020.01281)

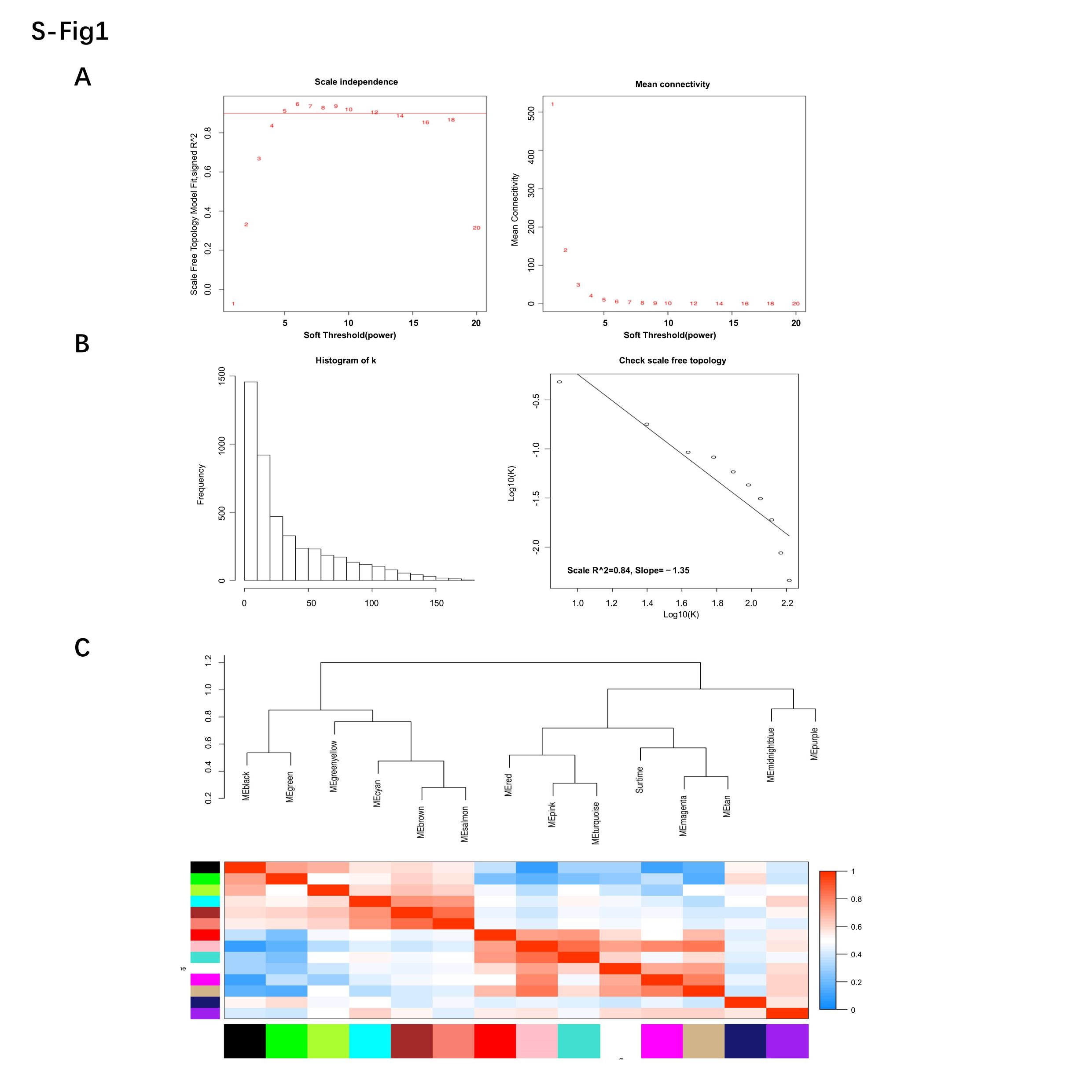

Supplement: Supplementary Figure 1 — (A) Network topology for different soft-thresholding powers. Numbers in the plots indicate the corresponding soft thresholding powers. The approximate scale-free topology can be attained at a soft-thresholding power of 5. (B) Assessing the scale-free topology when the soft-thresholding power was set to 5 (scale-free R2 = 0.84, slope = −1.35). (C) Eigengene adjacency heatmap. Different colors indicate the degree of correlation between modules. [file Image_1.JPEG]

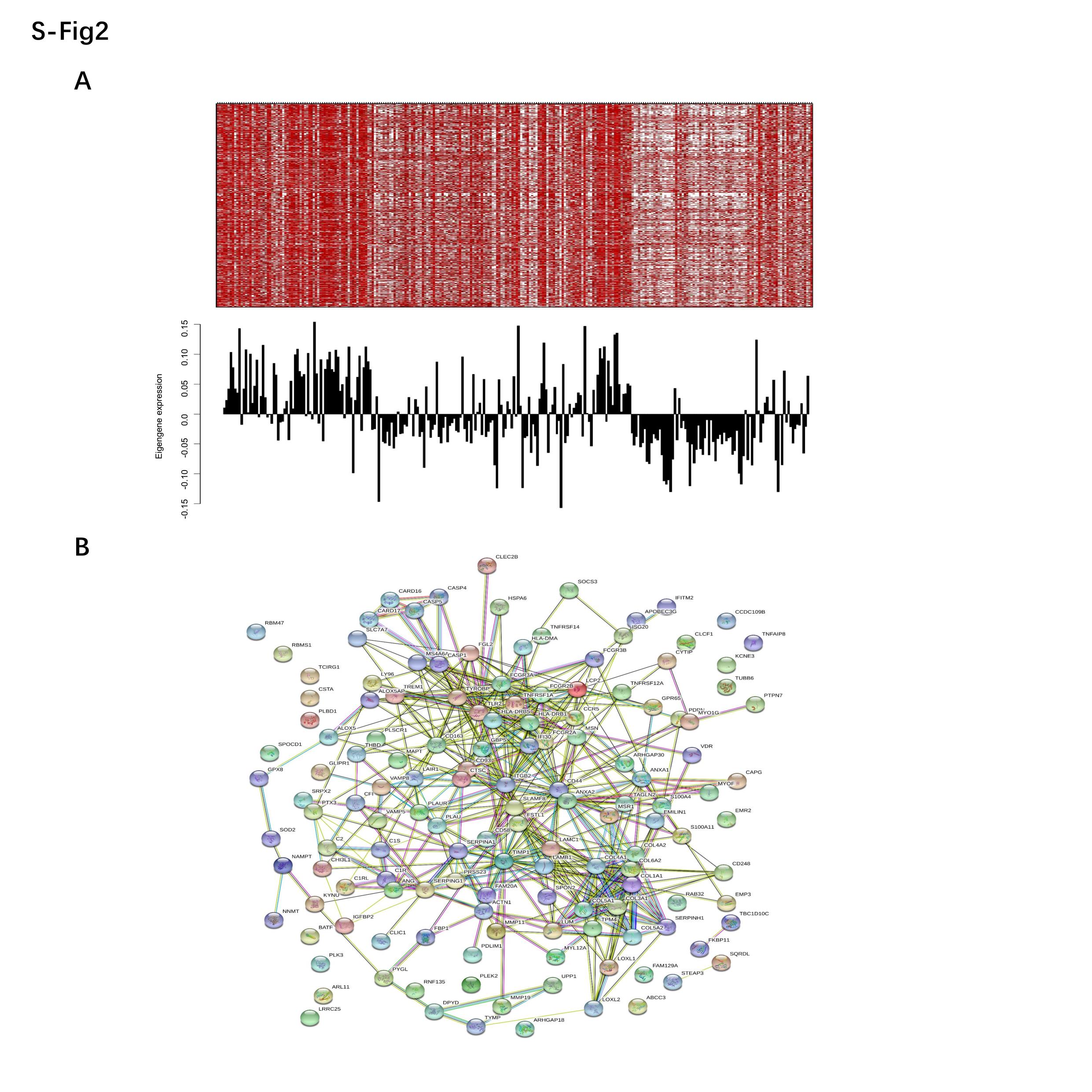

Supplement: Supplementary Figure 2 — (A) Heatmap for gene expression in black modules. The average expression of the eigenvectors of the black module is highly correlated with the expression of genes within the black module. (B) Protein–protein interaction network consisting of all genes in the black module. [file Image_2.JPEG]

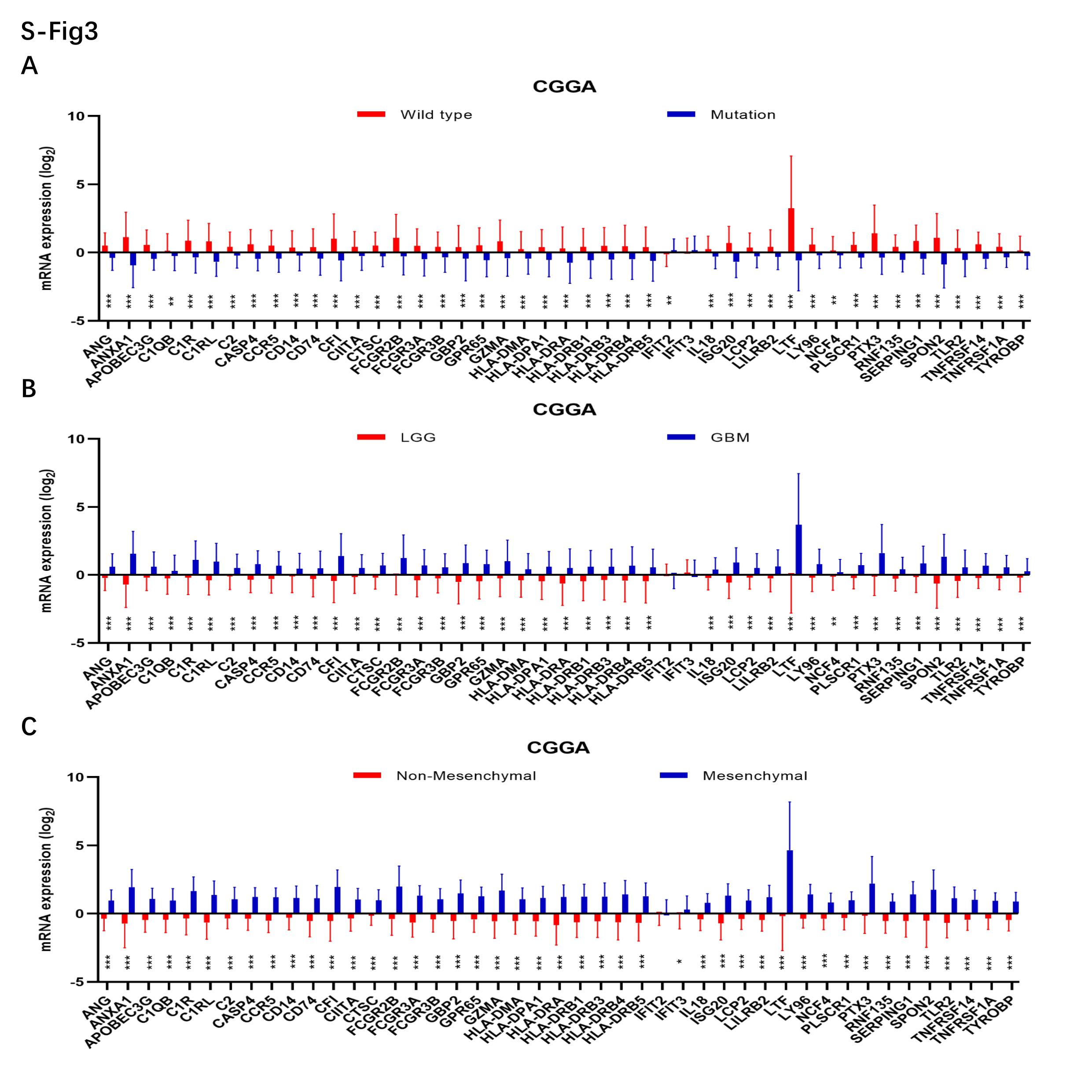

Supplement: Supplementary Figure 3 — The expression of intersection genes in different groups based on the CGGA database. (A) Expression of IDH wild-type and mutant. (B) Expression of LGG and GBM. (C) Expression of the non-mesenchymal subgroup and mesenchymal subgroup. [file Image_3.JPEG]

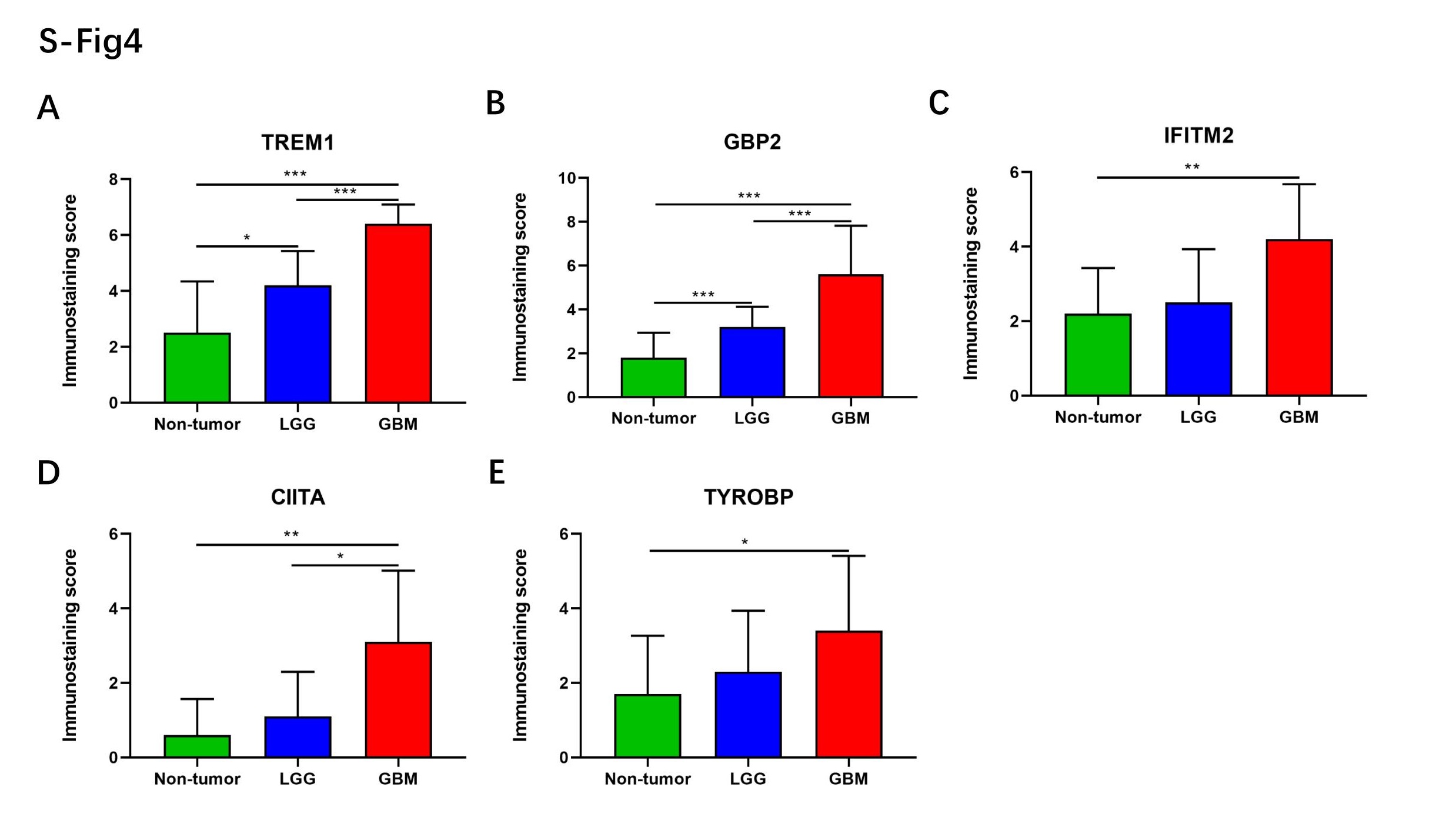

Supplement: Supplementary Figure 4 — Quantitative graph of IHC staining in normal tissue and glioma samples. (A) TREM1; (B) GBP2; (C) IFITM2; (D) CIITA; and (E) TYROBP. Significant difference between the two groups: *P < 0.05; **P < 0.01; ***P < 0.001. [file Image_4.JPEG]

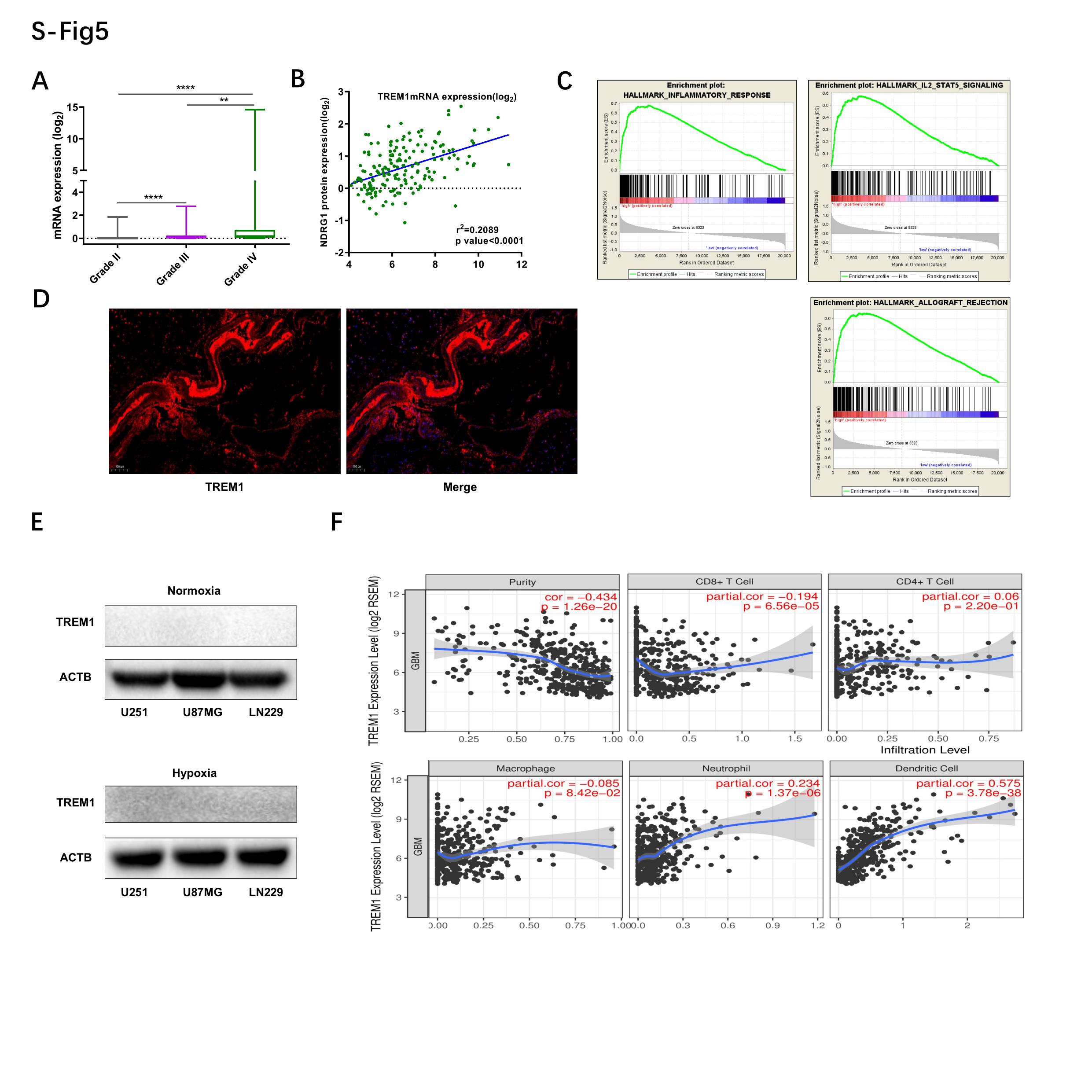

Supplement: Supplementary Figure 5 — (A) TREM1 mRNA expression levels in both LGG and GBM samples from the CGGA database. (B) Scatter plot displaying the correlation between TREM1 mRNA expression levels and NDRG1pT346 protein levels. (C) GSEA highlighting a positive association of increased TREM1 expression levels with inflammatory response, IL2-STAT5 signaling, and allograft rejection. (D) Immunofluorescence staining of GBM tissue sections. Red represents TREM1, blue represents DAPI (scale bar: 100 μm). (E) Western blot analysis of TREM1 protein levels in three GBM cell lines under normoxia and hypoxia. (F) The correlation between TREM1 mRNA expression and immune infiltration levels in GBM. These images were generated using TIMER. [file Image_5.JPEG]

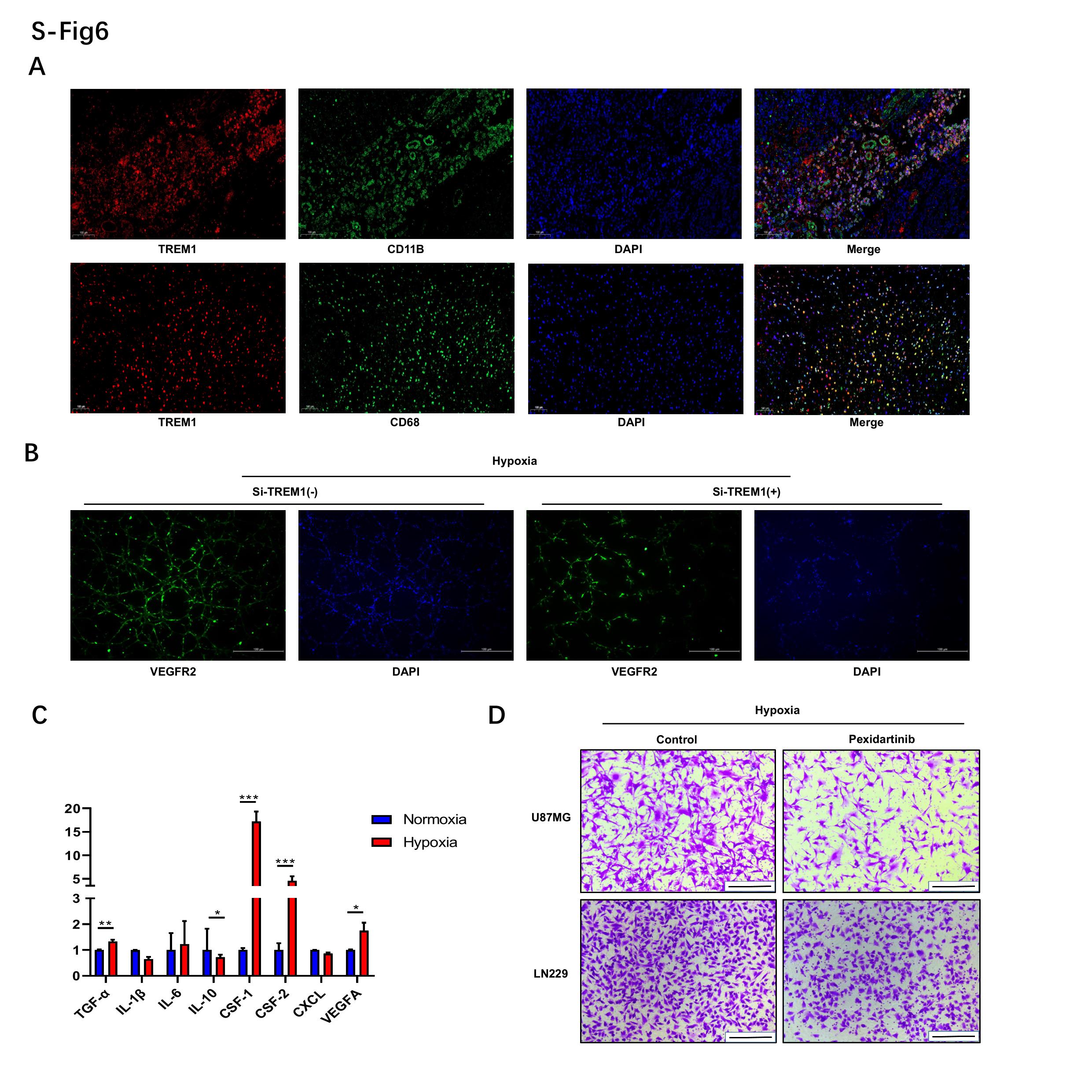

Supplement: Supplementary Figure 6 — (A) Immunofluorescence staining of GBM tissue sections. Red represents TREM1, green represents CD11b and CD68, respectively, and blue represents DAPI (scale bar: 100 μm). (B) Immunofluorescence staining of VM for U87MG under hypoxia condition plus control or si-TREM1. Green represents VEGFR2, and blue represents DAPI (scale bar: 100 μm). (C) mRNA expressions of TGF-α, IL1β, IL6, IL10, CSF1, CSF2, CXCL, and VEGFA were detected under normoxia and hypoxia. (D) Representative images of Transwell migration for U87MG and LN229 in both control and pexidartinib treatment under hypoxia (scale bar: 200 μm). Significant difference between the two groups: *P < 0.05; **P < 0.01; ***P < 0.001. [file Image_6.JPEG]
