# Supplementary material for: Identification of Immune-Related Genes Contributing to the Development of Glioblastoma Using Weighted Gene Co-expression Network Analysis
Source: Front Immunol. 2020 Jul 16;11:1281. doi: 10.3389/fimmu.2020.01281 (PMC7378359; doi:10.3389/fimmu.2020.01281)
Supplement: Supplementary Table 1 — Nomogram for predicting the proportion of glioma patients with OS based on the TCGA database. [file Table_1.docx]

**Supplementary
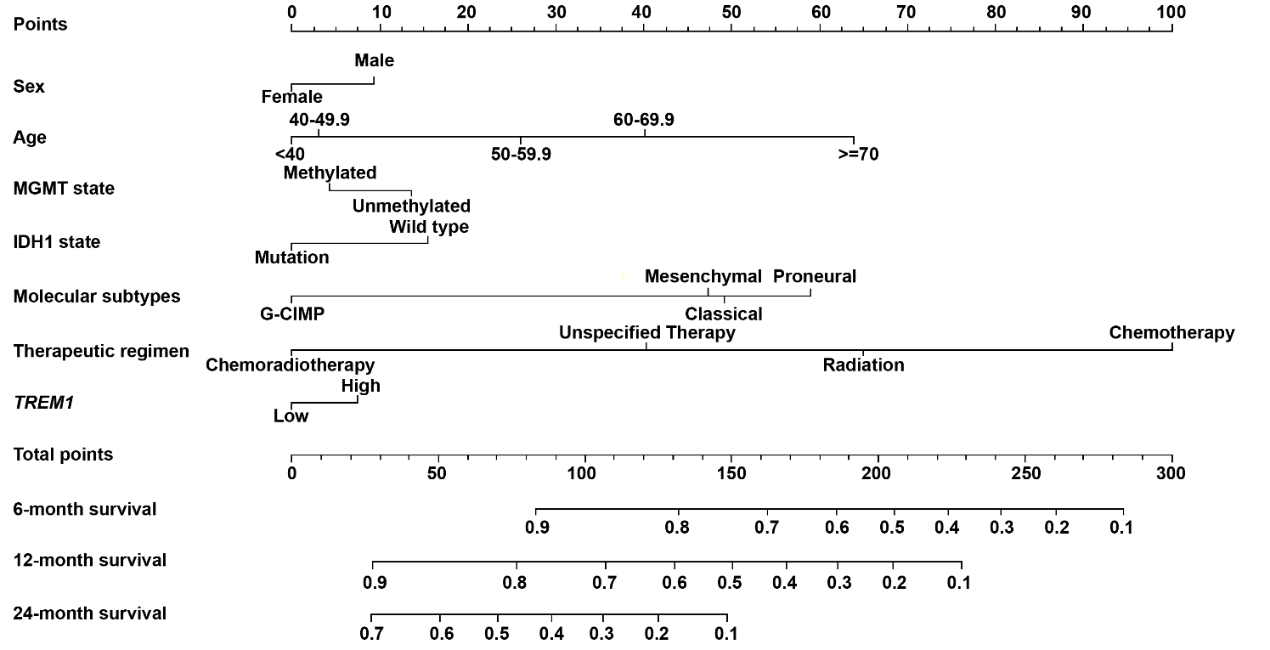
Table 1.** Nomogram for predicting the proportion of glioma patients with OS based on the TCGA database.
